# Identifying and profiling authoritative cardiology-related key opinion leaders on Xiaohongshu: a social media-based study

**DOI:** 10.3389/fpubh.2026.1889294

**Published:** 2026-07-27

**Authors:** Lili Wang, Qi Sun, Yang Wang, Tianyan Yu, Tingting Cai, Lingxiao Ye

**Affiliations:** 1 Department of Nursing, Ningbo Medical Center Lihuili Hospital, Ningbo, China; 2 Department of Cardiology, Ningbo Medical Center Lihuili Hospital, Ningbo, China; 3 Fudan University, Shanghai, China; 4 School of Nursing, Shanghai Donghai College, Shanghai, China; 5 Department of Anesthesiology, Fudan University Shanghai Cancer Center, Shanghai, China; 6 School of Nursing, Shanghai University of Traditional Chinese Medicine, Shanghai, China

**Keywords:** cardiovascular disease, digital health communication, key opinion leaders, social media, Xiaohongshu

## Abstract

**Objective:**

To identify and characterize distinct types of authoritative cardiology-related key opinion leaders (ACKOLs) on Xiaohongshu and examine their communication characteristics and engagement patterns.

**Methods:**

A social media profiling study was conducted on Xiaohongshu. Eligible ACKOLs were identified through manual searches and screened using predefined criteria. We applied a theory-informed 14-indicator profiling framework covering communicator credibility, social network visibility, message production, message expression and communication style, and audience engagement. K-means clustering was performed to classify ACKOLs, and word clouds were generated to visualize thematic characteristics.

**Results:**

A total of 150 ACKOL accounts comprising 46,616 posts were included. Four distinct types were identified: Public Health Educators, Clinical Narrators, Academic Interactors, and Authoritative Experts. Public Health Educators focused on accessible prevention-related science popularization content. Clinical Narrators emphasized clinical case sharing and psychosocial support. Academic Interactors showed the highest engagement and the strongest academic dissemination profile. Authoritative Experts had the largest follower base and strongest professional authority but relatively lower engagement. Together, these ACKOL types demonstrated complementary roles in information dissemination, emotional support, and audience engagement.

**Conclusion:**

ACKOLs on Xiaohongshu exhibit substantial heterogeneity in communication strategies and audience engagement. Different ACKOL types jointly form a complementary digital health communication ecosystem that may support cardiovascular disease management and patient-centered health communication.

## 1 Introduction

According to the 2025 report by the World Health Organization (WHO), approximately 19.8 million people die from cardiovascular diseases worldwide each year, accounting for nearly one-third of all deaths (1). Although substantial progress has been made in cardiovascular disease prevention and control across the continuum of care, including prevention, diagnosis, treatment, rehabilitation, and long-term management, numerous challenges remain in real-world implementation (2). The widespread adoption of early screening, pharmacological therapies, and device-based interventions has significantly reduced cardiovascular disease mortality. However, surgical treatment, long-term medication use, and their potential adverse effects and complications may continue to have a sustained impact on patients’ quality of life (3, 4). Across the disease trajectory, patients must sustain long-term lifestyle interventions, risk-factor control, and monitoring for complications. This process is challenging, and many patients find it difficult to maintain such changes over time (5).

Because cardiovascular diseases involve complex processes of behavioral management and psychosocial adaptation, patients and their families continue to face cognitive and emotional burdens during long-term treatment and adherence-related decision-making. Previous studies have shown that patients with cardiovascular diseases commonly experience multidimensional supportive care needs, including access to disease-related information, communication with healthcare professionals, treatment decision support, and guidance on managing side effects. When these needs remain unmet, they are often associated with lower quality of life and higher levels of anxiety and depression (6). Health inequities may further exacerbate disparities in resource access; for example, some patients may have difficulty obtaining timely specialist consultations or continuous follow-up support, thereby limiting their capacity for long-term health management (7, 8). Against this backdrop, institution-centered healthcare service models are unable to fully meet patients’ continuous and individualized needs across the entire disease trajectory.

With the development of digital health technologies and the rapid rise of social media in medical knowledge dissemination, how the public accesses health information is undergoing profound change. Before and after clinical consultations, patients actively search online for information related to diseases, medications, and treatments to help them understand clinical decisions and guide subsequent health behaviors (9–11). Meanwhile, online communities and health forums provide spaces for patients to share experiences and receive emotional support, enabling access to important supportive resources beyond the traditional healthcare system (12, 13). In this process, social media functions not only as a channel for information dissemination but also as an integrated health support platform that combines information acquisition, emotional communication, and behavioral guidance.

Within the social media health ecosystem, key opinion leaders (KOLs) have gradually become important intermediaries linking evidence-based medical knowledge, public understanding, and health behaviors. Studies have demonstrated that KOLs are important actors in public health information dissemination and health behavior promotion, with credibility derived from professional training, clinical experience, and sustained content output (14, 15). Unlike traditional medical services, KOLs can provide continuous information updates, experiential interpretation, and emotional responses in informal contexts, thereby partly compensating for limited physician–patient communication time and unequal access to medical resources. In long-term patient management, needs such as continuous information interpretation, supplementation of experiential knowledge, and emotional support often depend more heavily on informal health communication actors such as KOLs.

Existing studies on online health communication related to cardiovascular diseases have mainly focused on information quality assessment or single-dimensional communication metrics, such as views and likes. However, systematic analyses of the multidimensional roles of authoritative cardiology-related key opinion leaders (ACKOLs) in long-term patient management remain limited. Research has shown that patients with cardiovascular diseases rely heavily on online physicians and professional health information sources during disease management. In particular, patients often seek supplementary advice online regarding medication adjustment, symptom interpretation, and lifestyle management to compensate for the time and accessibility constraints of offline medical services (16–18). In addition, information provided by online professionals is considered substantially superior to nonprofessional sources in terms of credibility and decision support, making it relevant to the formation of patients’ health behaviors (19). Therefore, in social media contexts, cardiology KOLs with medical backgrounds and clinical experience essentially serve as a form of “extended medical support,” acting as an important bridge between the formal healthcare system and patients’ everyday health management.

Nevertheless, gaps remain in the existing literature. On the one hand, most studies have not distinguished among different types of cardiology KOLs in terms of their functions in information dissemination, emotional support, and behavioral guidance. Empirical analyses that systematically classify and compare them from multidimensional perspectives, such as content structure, audience engagement features, and language and communication style, are still lacking. On the other hand, current evidence is mainly derived from information-dissemination-oriented platforms such as Twitter/X, Facebook, or Weibo, whereas content-community platforms characterized by experience sharing and community interaction have received insufficient attention. In the Chinese context, health information dissemination is shaped not only by platform algorithmic mechanisms but also by culturally specific modes of expression and user interaction, which may further influence how cardiovascular health information is understood and adopted.

Xiaohongshu is a representative content-community platform in China that emphasizes authentic experience sharing and lifestyle-oriented expression. Its integration of images, text, and short videos allows professional knowledge to be presented in a more contextualized and intuitive manner (10). In cardiovascular disease management, this experience-based yet professionally informed mode of content expression may help patients better understand abstract medical knowledge and facilitate the translation of health information into behavioral change. Xiaohongshu therefore provides a representative research context for systematically analyzing the communication strategies of different types of cardiology KOLs and their roles in patient support.

Accordingly, this study focused on Xiaohongshu and constructed a multidimensional profiling framework combined with cluster analysis to identify different types of ACKOLs. It further examined their differences in background characteristics, content output, audience engagement features, and language and communication style. By exploring the functional positioning of different KOL types throughout the course of cardiovascular disease management and their ways of responding to patients’ multidimensional support needs, this study aimed to provide empirical evidence for developing stage-specific digital health communication strategies.

## 2 Methods

This study employed a multistage approach to profile authoritative cardiology-related key opinion leaders (ACKOLs) on Xiaohongshu. First, eligible accounts were identified through manual keyword searches and independently screened by two researchers according to predefined criteria for professional background, cardiology relevance, visibility, and noncommercial orientation. Second, the included accounts were characterized using a theory-informed 14-indicator profiling framework. The framework was used for quantitative description and clustering rather than as a psychometric scale or weighted screening tool. Before analysis, the collected data were systematically cleaned, standardized, and preprocessed to improve data quality and comparability. Subsequently, K-means clustering was applied to classify accounts, and the final cluster solution was evaluated using the elbow method, silhouette coefficient, and profile interpretability. Finally, cluster-specific word clouds were generated using Python to provide supplementary visualization of content focus.

### 2.1 Indicator selection for the conceptual profiling framework

To characterize ACKOLs on Xiaohongshu, we developed a theory-informed, literature-based profiling framework drawing on classic communication theory, McGuire’s input–output framework for persuasive communication, source credibility theory, and previous social media health influencer/KOL profiling studies (20, 21). The framework served as an operational tool for multidimensional quantitative profiling and clustering, rather than as a newly validated psychometric scale.

The selection of indicators was guided by the source-message-channel-receiver/effect logic of the communication process, including communicator characteristics, message production, communication style, and audience response. Background characteristics, including educational attainment, professional title, and hospital level, were used to reflect professional credibility and source authority. Social network visibility, represented by follower count, was used to capture online visibility and potential reach. Content output indicators, including posting frequency, science popularization proportion, and content format/content category composition, were used to describe message production and dissemination patterns. Message expression and communication style indicators, including readability index, dominant communication style, and positivity ratio, were used to capture how professional health information was expressed. Finally, audience engagement features, including average comments, average likes, average reposts, and engagement rate, were included to reflect audience response and the receiver/effect component of the communication process. The final framework included 14 indicators across communicator/message-side and audience-response layers. Its validity should therefore be interpreted as content and theoretical validity for social media profiling, not as evidence that the indicators measure a single latent construct.

### 2.2 Data collection

Data were collected through manual searches. In the initial stage, Chinese keyword equivalents of the following six terms were used to identify potential accounts: “heart,” “cardiovascular,” “cardiology,” “cardiac surgery,” “cardiocerebrovascular disease,” and “congenital heart disease.” All publicly available posts and interaction data from account creation to August 15, 2025, were retrieved, including post text, images, videos, likes, comments, and reposts. Two trained researchers independently reviewed all identified accounts and recorded usernames, content topics, and activity levels.

The inclusion criteria were as follows: (1) a verifiable professional background in medicine or health-related fields; (2) content focused on the prevention, treatment, care, rehabilitation, or patient support of cardiovascular diseases; and (3) a follower count of no fewer than 5,000. The exclusion criteria were as follows: (1) content unrelated to cardiology; and (2) commercial promotion that could undermine objectivity and professionalism. In this study, “authoritative” was operationalized primarily in relation to professional credibility and cardiology relevance, rather than audience engagement alone. Engagement indicators were analyzed only after account inclusion and were not used as evidence of scientific quality or clinical accuracy. Disagreements during screening were resolved through discussion. After the final list of ACKOLs was confirmed, all of their public content, including text, images, videos, and interaction data, was downloaded for subsequent analysis.

### 2.3 Data cleaning and coding

To ensure data quality, all downloaded data first underwent manual review. Two researchers independently examined the accounts and their posts, removing duplicate records, blank entries, and accounts or posts that were not primarily related to cardiovascular topics or were advertising-oriented. Account attributes and content categories were independently coded by the two researchers. Any disagreements were resolved through discussion, and historical posts or institutional affiliations were consulted when necessary to ensure classification accuracy and data credibility.

For dominant communication style, the coding unit was the individual post. Two trained researchers independently assessed the title, main text, visual captions, and overall communicative purpose. “Case storytelling” was coded when the post mainly described a patient case, clinical encounter, treatment trajectory, or experiential story with a narrative sequence. “Data interpretation” was coded when the post mainly explained research findings, clinical indicators, guidelines, statistics, mechanisms, diagnostic criteria, or treatment evidence. “Policy analysis” was coded when the post mainly interpreted health policies, institutional regulations, reimbursement issues, or public health policy implications. When a post contained multiple elements, the dominant style was assigned according to the component that occupied the largest proportion of meaningful content or represented the main communicative purpose of the post. If no single style was dominant, the post was coded as “mixed/other.” At the account level, dominant communication style was defined as the style accounting for the largest proportion of that account’s posts.

Readability was calculated on the basis of average sentence length and the proportion of technical terms and was used as a proxy measure of linguistic accessibility, with higher values indicating greater accessibility. Because readability does not directly measure comprehension, trust, perceived usefulness, or behavioral intention, it was interpreted cautiously as an indicator of how accessible the language was likely to be for general audiences. Positivity ratio was derived from sentiment analysis and represented the proportion of posts conveying positive emotions.

### 2.4 Data preprocessing

After manual cleaning, the retained data underwent technical preprocessing to support quantitative analysis. Specifically, post text was standardized by removing hyperlinks, special characters, and invalid tags. Categorical variables were converted into numerical codes and integrated into a structured database. Jieba word segmentation was then used to tokenize the text, and common stop words were removed to reduce noise and improve analytical precision.

### 2.5 User profiling and clustering

K-means clustering was used to classify ACKOLs into distinct subgroups based on the 14 profiling indicators. This method was selected because the study aimed to identify exploratory and interpretable account segments based on multiple standardized quantitative indicators, allowing accounts with similar multidimensional characteristics to be grouped into transparent and practically meaningful clusters. Before clustering, continuous variables were standardized to reduce the influence of different measurement scales, and categorical variables were converted into numerical codes according to predefined coding rules. No differential *a priori* weights were assigned to the indicators. To improve the robustness of the clustering results, the K-means algorithm was repeated multiple times with different initial cluster centers. Candidate cluster solutions were compared using the elbow method and silhouette coefficient. In addition to these statistical criteria, the interpretability and practical meaningfulness of the cluster profiles were also considered.

After cluster membership was determined, the characteristics of each cluster were examined by comparing patterns across the 14 indicators. Cluster labels were developed based on the dominant features of each subgroup, including professional background, content output, audience engagement, and communication style. Word clouds were subsequently generated for each cluster using Python to provide a supplementary visualization of the thematic focus.

## 3 Results

### 3.1 Overview of included ACKOLs

A total of 212 accounts were identified. After applying the inclusion and exclusion criteria, 150 authoritative cardiology-related key opinion leaders (ACKOLs) were included in the analysis, contributing 46,616 posts. Overall, these ACKOLs differed substantially in follower count, posting frequency, content structure, and engagement level. For example, follower count ranged from 5,000 to more than 100,000, and average monthly posting frequency ranged from fewer than 5 posts to more than 50 posts. Some accounts primarily focused on disease prevention and science popularization content, whereas others emphasized clinical case content or psychosocial support content. In terms of engagement, the distributions of average likes, comments, and reposts varied widely, demonstrating marked diversity in digital health communication.

### 3.2 Cluster analysis

**Figure 1:**
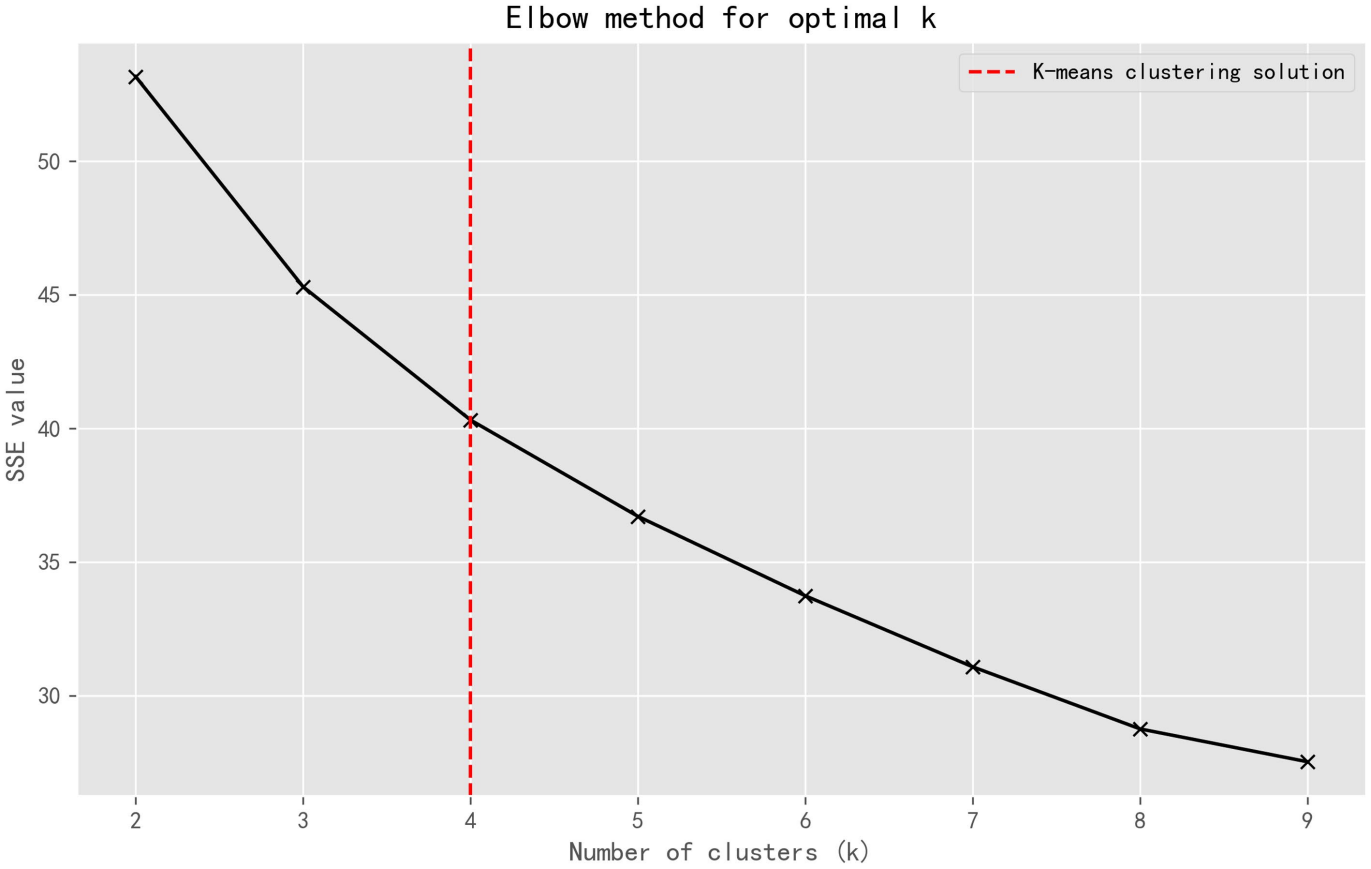
Elbow plot for determining the candidate cluster solutions of K-means clusters. The within-cluster sum of squares decreased markedly until K = 4 and then declined more gradually, suggesting the acceptability of a four-cluster solution when considered together with profile interpretability.

Based on the 14 profiling indicators, K-means clustering analysis was conducted on the 150 ACKOLs. Several cluster solutions were examined to determine the optimal number of clusters. As shown in Figure 1, as the number of clusters (K) increased, the within-cluster sum of squares (SSE) showed a downward trend. The decrease was more pronounced from K = 2 to K = 4, after which the rate of decline became more gradual, suggesting a potential elbow point at K = 4.

**Figure 2:**
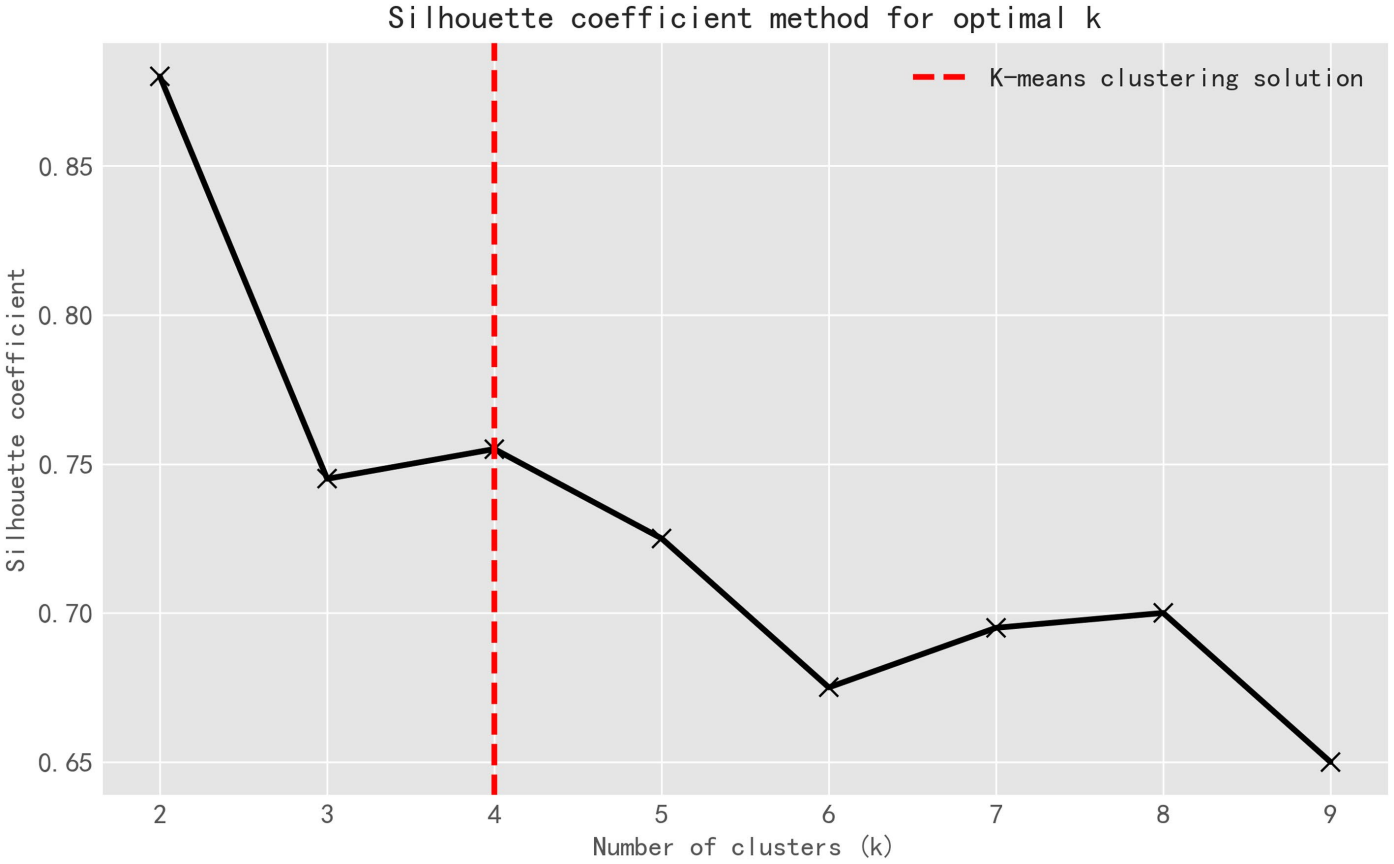
Silhouette coefficient plot across candidate cluster numbers. Although K = 2 showed the highest average silhouette coefficient, K = 4 maintained acceptable cohesion and separation while providing a more interpretable ACKOL classification.

As shown in Figure 2, the average silhouette coefficient was highest when K = 2, indicating strong separation for a two-cluster solution. However, the two-cluster solution was considered too coarse to capture the multidimensional heterogeneity of ACKOLs in terms of professional background, content output, audience engagement, and communication style. The silhouette coefficient for K = 4 remained acceptable, at approximately 0.75, indicating adequate cluster cohesion and separation. Moreover, the four-cluster solution provided more meaningful and interpretable subgroup profiles. Therefore, considering both statistical indicators and substantive interpretability, K = 4 was selected for the final clustering solution.

### 3.3 Profiles of the four ACKOL types

**Figure 3:**
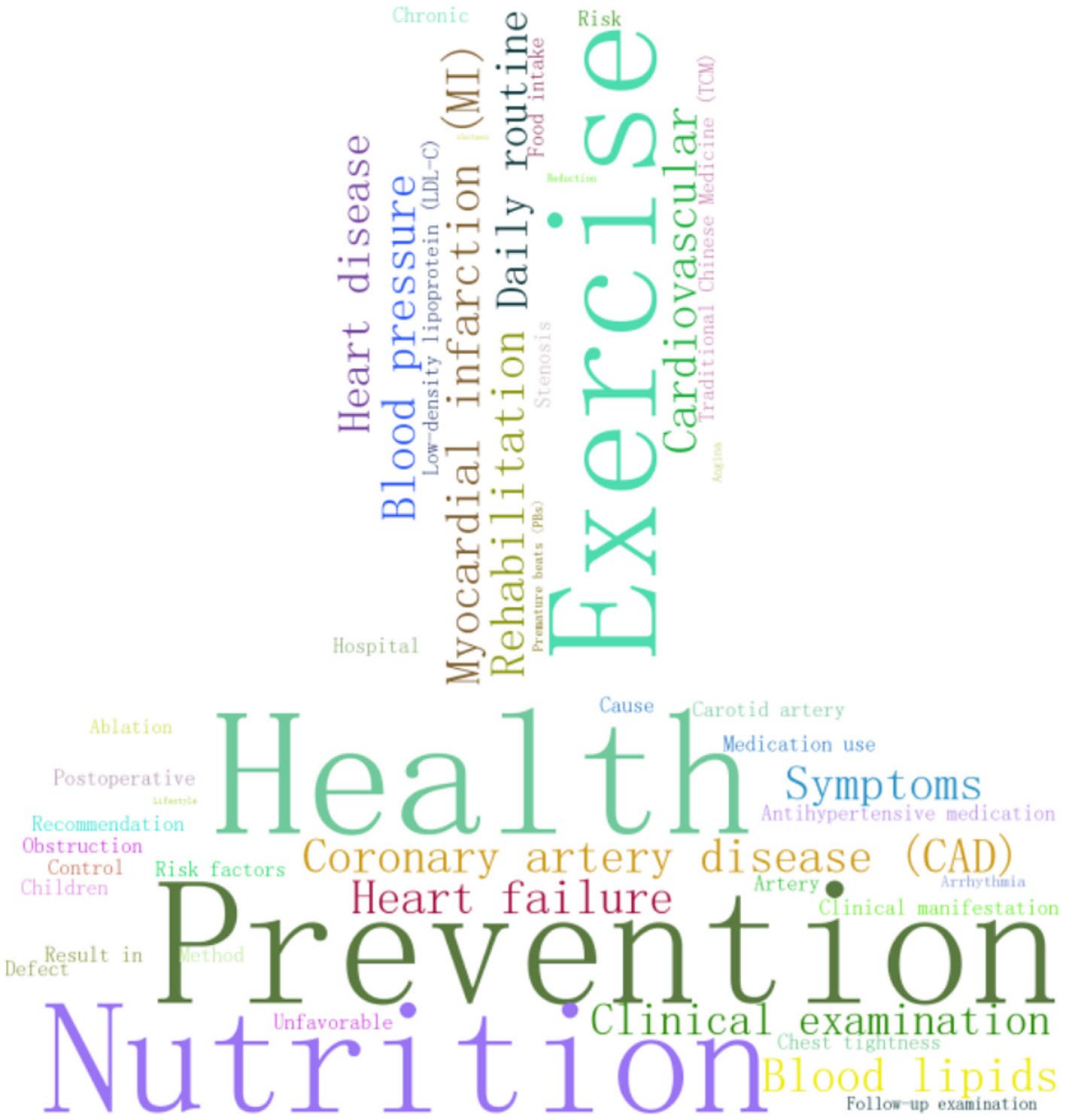
Word cloud of Public Health Educators, highlighting frequent prevention- and lifestyle-oriented terms.

**Figure 4:**
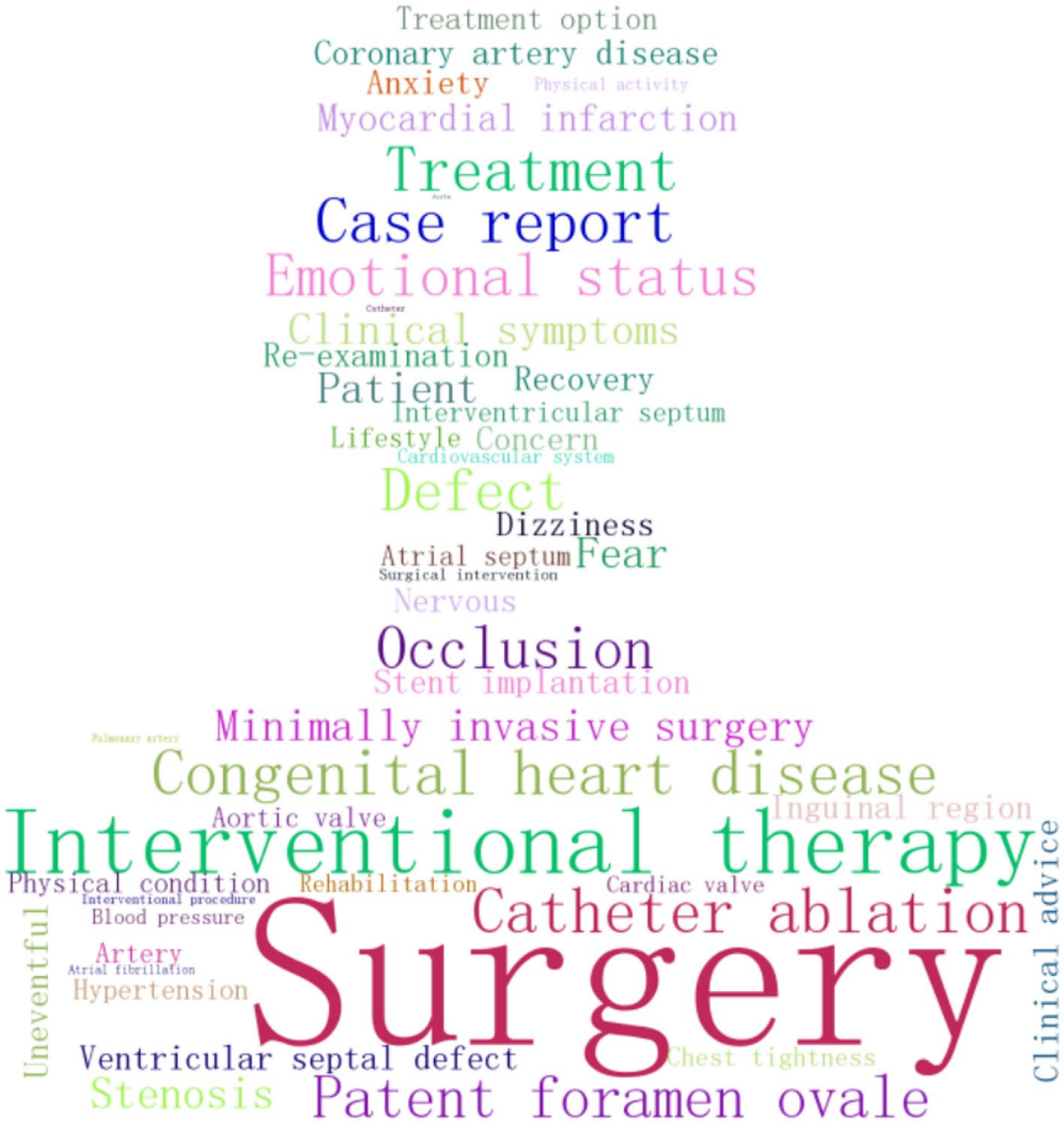
Word cloud of Clinical Narrators, highlighting clinical case, treatment, and psychosocial-support terms.

**Figure 5:**
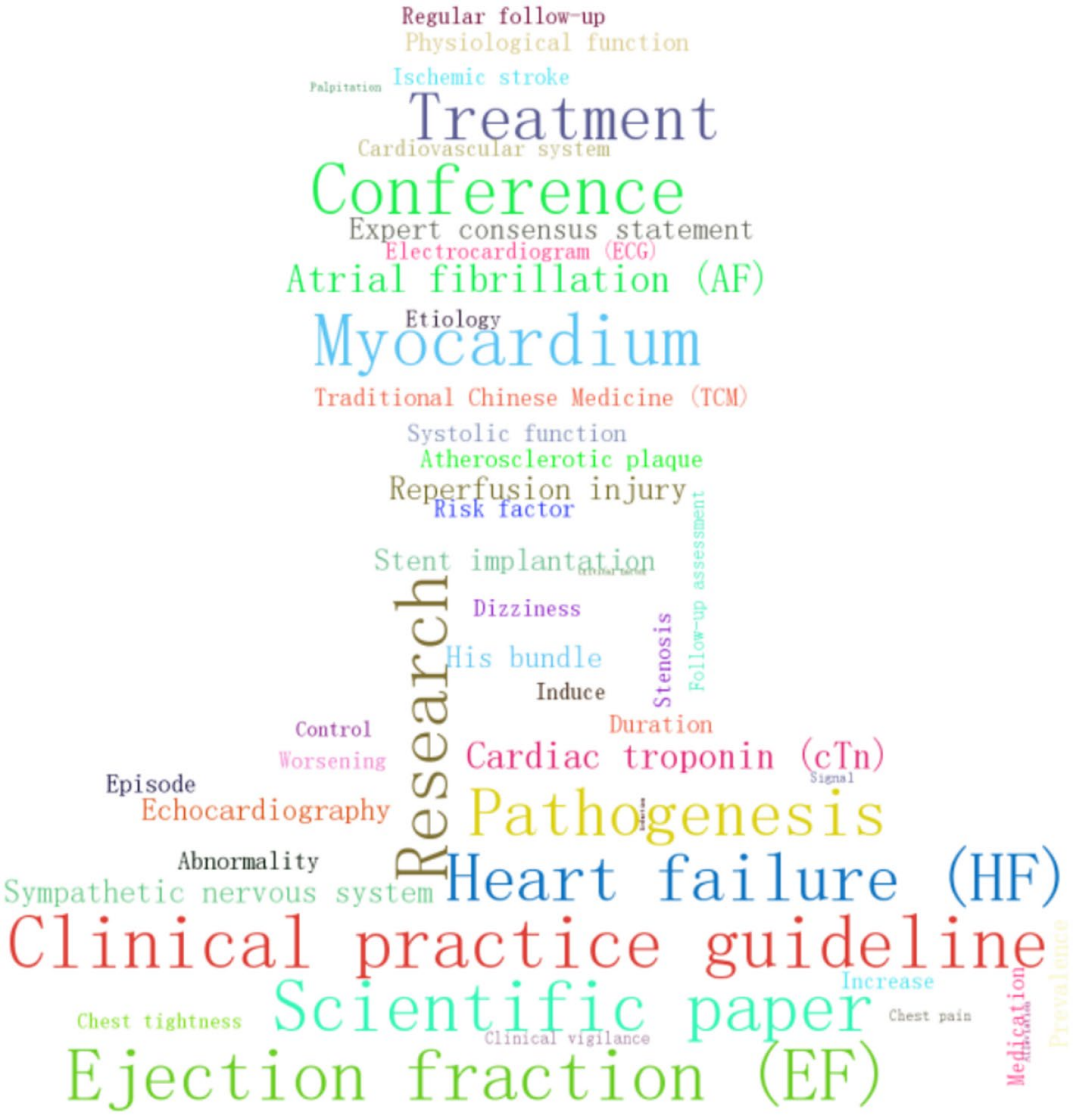
Word cloud of Academic Interactors, highlighting academic dissemination, guideline, and research-related terms.

**Figure 6:**
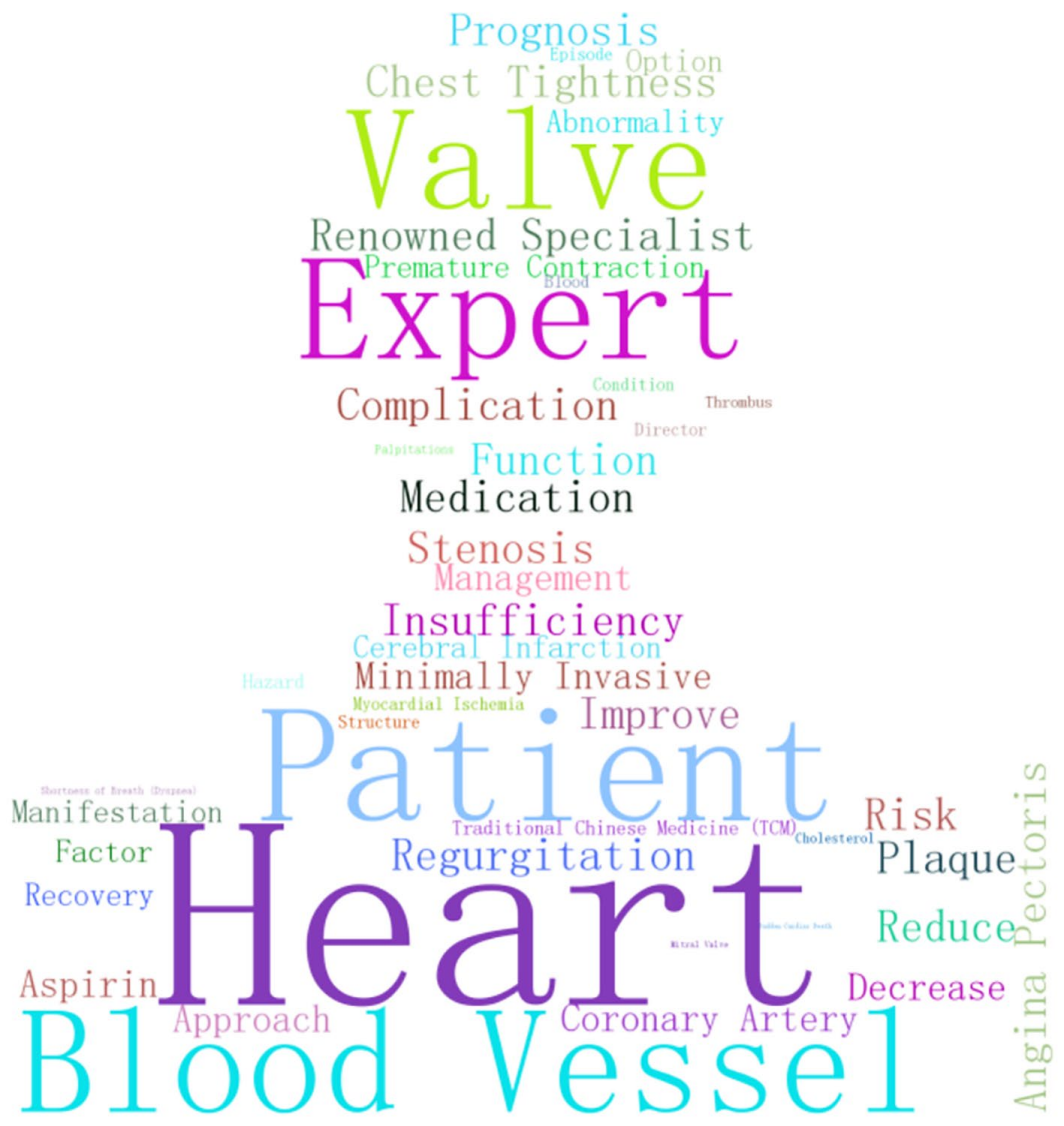
Word cloud of Authoritative Experts, highlighting authority-based clinical and disease-management terms.

The included ACKOLs were classified into four types: Public Health Educators, Clinical Narrators, Academic Interactors, and Authoritative Experts. These groups showed clear differences in background characteristics, content output, audience engagement features, and language and communication style (Tables 1, 2). Word clouds were generated for each group to visualize high-frequency terms and highlight thematic emphases (Figures 3–6).

**Table 1:** Profiling framework for authoritative cardiology-related key opinion leaders (ACKOLs) on Xiaohongshu.

Dimension	Indicator	Definition & description	Data type
Communicator credibility	Educational attainment	Highest academic qualification of the KOL	Categorical
	Professional title	Clinical rank	Categorical
	Hospital level	Affiliated hospital level of the KOL	Categorical
Social network visibility	Follower count	Number of followers, reflecting popularity and online visibility	Continuous
Message production	Posting frequency	Average number of posts/month	Continuous
	Science popularization proportion	Percentage of posts devoted to public health education	Continuous (%)
	Content category composition	Proportions of posts in each predefined thematic content category	Continuous (%)
Audience engagement	Average comments	Mean number of comments received per post	Continuous
	Average likes	Mean number of likes received per post	Continuous
	Average reposts	Mean number of reposts per post	Continuous
	Engagement rate	(Likes + Comments + Reposts) / Followers	Continuous (%)
Expression and style	Readability index	Readability score calculated from average sentence length and proportion of technical terms	Continuous
	Dominant communication style	Dominant communication style of the post (case storytelling, data interpretation, or policy analysis)	Categorical
	Positivity ratio	Proportion of posts conveying positive emotions, derived from sentiment analysis	Continuous (%)

**Table 2:** Profiles of authoritative cardiology-related key opinion leaders (*n* = 150).

Public health educators		
Type description	Highest proportion of science popularization content (approximately 64%); mainly focused on simplified content (disease prevention, diet, and exercise)	
Communicator credibility	Educational attainment	PhD: 33%
	Professional title	Chief physician: 15%
	Hospital level	Tertiary hospital affiliation: 76%
Social network visibility	Follower Count	11,137
Message production	Posting frequency	22 posts/month
	Science popularization proportion	64%
	Content format/category	Academic dissemination: 1%;
		Clinical case content: 5%;
		Rehabilitation and lifestyle content: 24%;
		Psychosocial support content: 9%
Audience engagement	Average comments	5
	Average likes	76
	Average reposts	41
	Engagement rate	3.9%
Expression and Style	Readability index	0.4
	Dominant communication style	Data interpretation
	Positivity ratio	31%

Clinical narrators		
Type description	Clinical case sharing (22%, highest among clusters); psychosocial support content (26%, highest among clusters); case storytelling	
Communicator credibility	Educational attainment	PhD: 39%
	Professional title	Chief physician: 31%
	Hospital level	Tertiary hospital affiliation: 92%
Social network visibility	Follower count	14,046
Message production	Posting frequency	21 posts/month
	Science popularization proportion	46%
	Content format/category	Academic dissemination: 2%;
		Clinical case content: 22%;
		Rehabilitation and lifestyle content: 11%; Psychosocial support content: 26%
Audience engagement	Average comments	20
	Average likes	117
	Average reposts	29
	Engagement rate	4.1%
Expression and style	Readability index	0.4
	Dominant communication style	Case storytelling
	Positivity ratio	33%

Academic Interactors		
Type description	Academic dissemination (6%, highest among clusters); engagement rate (5.1%, highest among clusters); lowest readability index (0.3), indicating highly technical expression	
Communicator credibility	Educational attainment	PhD: 47%
	Professional title	Chief physician: 19%
	Hospital level	Tertiary hospital affiliation: 88%
Social network visibility	Follower count	16,687
Message production	Posting frequency	24 posts/month
	Science popularization proportion	55%
	Content format/category	Academic dissemination: 6%;
		Clinical case content: 11%;
		Rehabilitation and lifestyle content: 21%;
		Psychosocial support content: 17%
Audience Engagement	Average comments	30
	Average likes	143
	Average reposts	79
	Engagement rate	5.1%
Expression and style	Readability index	0.3
	Dominant communication style	Data interpretation
	Positivity ratio	41%

Authoritative experts		
Type description	Largest follower base (approximately 27,000); predominantly senior physicians (92% chief physicians); focused on disease treatment and complication management	
Communicator credibility	Educational attainment	PhD: 44%
	Professional title	Chief physician: 92%
	Hospital level	Tertiary hospital affiliation: 86%
Social network visibility	Follower count	27,084
Message production	Posting frequency	24 posts/month
	Science popularization proportion	60%
	Content format/category	Academic dissemination: 4%;
		Clinical case content: 11%;
		Rehabilitation and lifestyle content: 21%; Psychosocial support content: 15%
Audience engagement	Average comments	16
	Average likes	209
	Average reposts	96
	Engagement rate	3.4%
Expression and style	Readability index	0.5
	Dominant communication style	Data interpretation
	Positivity ratio	31%

#### 3.3.1 Type 1: public health educators (*n* = 46)

This group had the smallest follower count, with a mean of approximately 11,137 followers, but the highest science popularization proportion (64%). Video-based posts accounted for almost all content (94%). Posts mainly presented simplified information, including disease prevention, diet, and exercise, with particular attention to coronary heart disease, myocardial infarction, and heart failure. Engagement was moderate, with an engagement rate of 3.9%, whereas average comments were relatively low, with a mean of 5 comments per post. The readability index was 0.4, indicating relatively accessible language, and the dominant communication style was data interpretation. This type of ACKOL provides the public with easy-to-understand science popularization content, although it is relatively limited in professional depth and audience retention. The word cloud showed frequent occurrences of terms such as “prevention,” “exercise,” and “diet,” highlighting this group’s thematic emphasis on public health education (Figure 3).

#### 3.3.2 Type 2: clinical narrators (*n* = 36)

This group had a moderate follower count, with a mean of approximately 14,046 followers. It had the highest proportion of clinical case content (22%) and the highest proportion of psychosocial support content (26%). The engagement rate was 4.1%, and average comments reached 20 comments per post, indicating strong audience resonance. The readability index was 0.4, suggesting moderate content complexity, and the dominant communication style was case storytelling. Posts often focused on congenital heart disease and patent foramen ovale, integrating clinical expertise with patient communication. This type of ACKOL strikes a balance between clinical knowledge transmission and humanistic care, providing patients with both informational and psychosocial support. The word cloud highlighted keywords such as “treatment,” “surgery,” “intervention,” and “mood,” reflecting the coexistence of clinical knowledge and humanistic care in this group’s content (Figure 4).

#### 3.3.3 Type 3: academic interactors (*n* = 32)

This group had a moderate follower count, with a mean of approximately 16,687 followers. It showed the highest posting frequency, at 24 posts per month, and the highest engagement rate, at 5.1%. Each post received an average of 143 likes, 30 comments, and 79 reposts. The science popularization proportion was 55%, while academic dissemination content reached the highest proportion among all groups (6%). This group had the lowest readability index (0.3), indicating the densest use of professional terminology and relatively lower overall comprehensibility. The dominant communication style was data interpretation. This type of ACKOL combines academic dissemination with active engagement and demonstrates notable performance in both professional influence and audience engagement. The word cloud contained academic terms such as “guidelines,” “research,” “myocardium,” “paper,” “ejection fraction,” and “pathological mechanisms,” as well as disease-related terms such as “heart failure,” highlighting this group’s focus on academic dissemination supplemented by selective disease discussion (Figure 5).

#### 3.3.4 Type 4: authoritative experts (*n* = 36)

This group had the largest follower count, with a mean of approximately 27,084 followers, and the highest proportion of physicians with senior professional titles, with chief physicians accounting for 92%. Its content was mainly science-popularization oriented, with a science popularization proportion of 60%, and also included clinical case content (11%) and rehabilitation and lifestyle content (21%). The engagement rate was 3.4%, and average comments were 16 comments per post, higher than that of Type 1 but lower than those of Types 2 and 3. This group had the highest readability index (0.5), indicating simpler and more understandable language. Posts frequently focused on disease treatment and complication management, including valvular heart disease, vascular stenosis, and complication prevention and management, reflecting an emphasis on the authoritative dissemination of professional knowledge. Drawing on their professional background and senior titles, these ACKOLs have substantial influence in disseminating clinical and science popularization knowledge related to cardiovascular diseases, although their engagement with audiences remains relatively limited. The word cloud highlighted keywords such as “expert” and “renowned doctor,” underscoring knowledge dissemination based on professional authority (Figure 6).

Overall, the four types of ACKOLs showed marked differences in audience reach, content orientation, audience engagement features, and language and communication style. Public Health Educators emphasized accessible prevention and lifestyle guidance; Clinical Narrators combined clinical case content with psychosocial support content; Academic Interactors integrated academic dissemination with active engagement; and Authoritative Experts relied on professional identity for knowledge dissemination but showed relatively limited audience engagement. Together, these distinct profiles highlight the diverse and complementary roles of ACKOLs in cardiovascular health communication and provide a basis for further examining their contributions to patient education and digital health promotion.

## 4 Discussion

Based on the characteristics of authoritative cardiology-related key opinion leaders on Xiaohongshu, this study constructed a theory-informed profiling framework comprising 14 indicators across communicator/message-side and audience-response layers. Cluster analysis further identified four distinct types of ACKOLs: Public Health Educators, Clinical Narrators, Academic Interactors, and Authoritative Experts. Compared with previous studies that relied mainly on single indicators, such as follower count (22), this study integrated multidimensional attributes, including professional credibility, social network visibility, message production, audience engagement features, and message expression and communication style, thereby enabling a more refined and context-specific typological classification of health-related KOLs. Different types of ACKOLs do not exist in isolation within digital health communication; rather, they jointly constitute a multilayered information-support system that may respond to patients’ multidimensional needs for information acquisition, emotional support, and long-term self-management. Compared with the Weibo-based study of oncology-related KOLs, the classification proposed in this study shows certain functional correspondence with the “expert knowledge,” “popular science knowledge,” “emotional support,” and “social interaction” types identified by Duan et al. (21), suggesting that health communication roles may share commonalities across different platforms.

The four types of ACKOLs together reveal a structural tension, namely, the dynamic balance among information accessibility, professional authority, and audience engagement. Through their distinctive yet complementary communication roles, they may address different dimensions of patients’ health information needs. Public Health Educators were characterized by simplified and visualized expression; Authoritative Experts by professional credibility; Academic Interactors by knowledge depth and interactive dissemination; and Clinical Narrators by contextualized narratives and emotional connection. These four roles are functionally complementary and jointly form a digital health communication ecosystem that covers different cognitive levels and emotional needs.

Compared with other types, Public Health Educators mainly served as an “entry point” for basic knowledge dissemination and health literacy improvement. With the highest science popularization proportion (64%), the dominant use of video format (94%), and accessible language (readability index: 0.4), this type focused on public health literacy and disease-prevention awareness. These accounts translated complex medical knowledge into action-oriented guidance centered on “prevention,” “diet,” and “exercise.” This strategy is consistent with the principle of “content accessibility” emphasized in recent health communication research, which argues that visualized and lifestyle-oriented content can lower the threshold for understanding health information, improve public health literacy and self-efficacy, and serve broader populations during the stages of prevention and health promotion (23). However, compared with types that emphasize deeper interaction or emotional connection, this communication model tended to rely more on one-way information transmission and may therefore have limitations in sustaining engagement.

Compared with accessibility-oriented Public Health Educators, Authoritative Experts placed greater emphasis on information authority and professional credibility, particularly in disease treatment, long-term management, guideline interpretation, and sustained care. These findings are similar to those of Zou et al. (24), suggesting that differences in professional background are associated with different positioning and dissemination pathways of health communication content on Chinese social media. This ACKOL type (*n* = 36) had the largest follower count, the highest proportion of chief physicians (92%), and the highest readability index (0.5). However, its engagement rate (3.4%) was the lowest among the four groups, and its average comments per post (16) were lower than those of Clinical Narrators and Academic Interactors, indicating a paradox in which authority coexisted with weaker interaction. This phenomenon may be related to the continuation of the traditional clinical “one-to-many” education model, which lacks the bidirectional interaction mechanisms required in social media environments (25). This pattern is consistent with Chen et al.’s analysis of cancer communication on Chinese social media, which found that accounts with strong professional identities have advantages in establishing initial trust but may struggle to promote deeper engagement when interaction design is insufficient (26). Weng et al. (27), in a scoping review of patient opinion leaders, noted that effective patient opinion leaders require not only professional knowledge and experience but also communication capacity, broad influence, and active responsiveness. These findings suggest that although professional authority constitutes the foundation of communication, user engagement is also critical for improving communication effectiveness.

By contrast, Academic Interactors and Clinical Narrators were characterized by communication strategies that may facilitate more active information processing. Academic Interactors had the highest proportion of academic dissemination content (6%) and the highest engagement rate (5.1%). Chen et al. (28)’s study of leaders in the pediatric COVID-19 vaccination campaign on Twitter showed that communicators with clinical expertise had greater influence within social networks, suggesting that the high engagement rate of this ACKOL type may reflect the appeal of professional authority to specific audience groups. These audiences may have relatively high health literacy or professional backgrounds and may therefore be more willing to engage with academic content. The dense appearance of terms such as “guidelines,” “research,” “myocardium,” “paper,” “ejection fraction,” and “pathological mechanisms” in the word cloud confirms the academic dissemination orientation of this type. For patients who need an in-depth understanding of disease mechanisms or who follow the latest research progress, especially patients managing chronic diseases over the long term and their family members, Academic Interactors provide a depth of knowledge that traditional clinical consultations often cannot fully cover. Their lower readability index (0.3) also reflects the potential tension between professional depth and communicative accessibility.

Clinical Narrators were characterized by an empathetic narrative environment, reflected in the highest proportions of clinical case content (22%) and psychosocial support content (26%), as well as a case storytelling style. This type reflects the “community DNA” of Xiaohongshu, where users are more inclined to establish emotional connections through real-life cases (29). This pattern is consistent with narrative health communication theory, which holds that storytelling based on personal experiences can enhance emotional engagement and increase the perceived relevance of health information (30). Worsdale et al.’s (23) study showed, in the context of endometriosis screening, that emotional and informational support provided through personal experience-based narratives can enhance self-efficacy and positive emotions (31), allowing participants to obtain both knowledge and psychosocial support and further promoting screening intention. Studies have shown that patients with cardiovascular diseases are prone to adverse psychological problems such as anxiety and depression (31, 32). If negative emotions are not identified and addressed in a timely manner, they may not only impair quality of life but also contribute to adverse cardiovascular events and hinder rehabilitation. The combination of frequent clinical case content and psychosocial support content among Clinical Narrators may transmit medical information while strengthening cardiovascular patients’ engagement through emotional connection, which may explain this group’s highest average number of comments per post (20 comments/post). This suggests that clinical narrative may serve not only as an information dissemination strategy but also as a potential tool for patient empowerment. Psychosocial support content should therefore be integrated into clinical information communication to address the often-overlooked psychological needs of patients with cardiovascular diseases. This also indicates that the role of medical KOLs on social media is not determined solely by professional authority or follower count, but by their ability to balance professional knowledge, information accessibility, and audience engagement, which is consistent with the argument proposed by Wu et al. (33).

Overall, this functional differentiation centered on “accessibility-authority-engagement” reveals the division of roles among different types of ACKOLs in digital health communication for cardiovascular diseases. Specifically, through differences in information expression, professional depth, and audience engagement features, each KOL type may correspond to patients’ core needs at different stages of disease, thereby forming a hierarchical support system. This structure is consistent with the characteristics of long-term cardiovascular disease management. Studies have shown that patients with chronic cardiovascular diseases, such as coronary heart disease and heart failure, face continuous needs for information acquisition, psychosocial support, and self-management, which are often difficult to fully satisfy through traditional healthcare services alone (34). Against this background, social media platforms provide an important supportive field for medical professionals and authoritative opinion leaders, enabling them to provide continuous information interpretation and psychosocial support beyond clinical settings. Existing studies have also shown that informational and emotional interaction in online health communities can improve patient engagement and self-management capacity (35, 36), while social support is an important factor influencing self-care adherence and quality of life among patients with cardiovascular diseases (37). Therefore, the findings of this study further suggest that different types of ACKOLs may jointly construct a digital health communication ecosystem that may support patients’ long-term disease management through differentiated configurations of communication strategies.

From a theoretical perspective, these findings also have implications for health communication research, although they should be interpreted within the specific context of ACKOLs on Xiaohongshu. Rather than proposing a new general communication theory, by operationalizes the source-message-receiver/effect logic in platform-based cardiovascular health communication, this study suggests that medical KOLs should be understood not only through source credibility, such as professional credentials and institutional affiliation, but also through message production, expression style, and audience response. In addition, the four ACKOL types show that professional authority, communicative accessibility, and audience engagement do not necessarily align. This finding refines the application of source credibility theory in social media health communication and provides an empirical typology for understanding different configurations of accessibility, authority, academic orientation, narrative expression, and engagement among cardiology-related KOLs on Xiaohongshu.

The platform-specific context should also be emphasized. Xiaohongshu is characterized by lifestyle-oriented expression, experience sharing, multimodal presentation, and community interaction. These platform affordances may shape how medical professionals present cardiovascular health information and how users engage with such content. Therefore, the ACKOL profiles identified here should be interpreted within the specific context of Xiaohongshu. The same profiling framework may provide a useful reference for future comparative studies across platforms and for developing platform-sensitive digital public health communication strategies.

### 4.1 Limitations

This study has several limitations. First, the data were derived solely from Xiaohongshu. The identified ACKOL profiles may not be directly generalizable to other platforms such as Weibo, WeChat, Douyin, TikTok, Twitter/X, or Facebook, where user demographics, algorithmic recommendation mechanisms, content formats, and interaction norms may differ. For example, short-video platforms may favor highly visual and emotionally engaging content, whereas microblogging platforms may facilitate broader public discussion and rapid information diffusion. Therefore, the theoretical implications of this study should also be interpreted cautiously as context-specific insights derived from ACKOL communication on Xiaohongshu, rather than as universally applicable conclusions for all health communication platforms, disease areas, or KOL populations. Second, the analysis focused mainly on observable content characteristics and engagement metrics, which may not fully capture the complexity of multimodal communication strategies or the contextual factors that shape user engagement. Third, engagement metrics such as likes, comments, reposts, and engagement rate reflect audience response and communication reach but do not directly indicate the scientific quality, evidence basis, clinical accuracy, or completeness of health information. This study did not conduct a formal expert appraisal or evidence-based quality assessment of each post. Fourth, the cross-sectional and observational design precludes causal inference between KOL characteristics and audience engagement or between exposure to ACKOL content and changes in patients’ knowledge, attitudes, decision-making, or health behaviors. Future research could adopt multi-platform, longitudinal, and user-centered designs and combine social media analytics with expert content appraisal to examine the accuracy, completeness, clinical reliability, and real-world effects of health information disseminated by medical KOLs.

## 5 Conclusion

This study identified four distinct types of authoritative cardiology-related key opinion leaders on Xiaohongshu: Public Health Educators, Clinical Narrators, Academic Interactors, and Authoritative Experts. These types differed substantially in content orientation, communication approach, and audience engagement, reflecting a functionally differentiated structure within digital health communication. Public Health Educators focused on disseminating basic prevention-related information; Clinical Narrators strengthened psychosocial support content and patient resonance; Academic Interactors combined professional knowledge dissemination with high engagement; and Authoritative Experts enhanced information credibility but showed relatively lower engagement. Together, these findings provide a context-specific empirical typology of ACKOLs on Xiaohongshu and suggest that the complementary roles of different KOL types may inform more targeted and platform-sensitive digital health communication strategies.

## Data Availability

The data supporting the findings of this study are available from the corresponding author upon reasonable request, subject to appropriate restrictions to protect user privacy and comply with platform data policies.
